# Using tweets to understand changes in the spatial crime distribution for hockey events in Vancouver

**DOI:** 10.1111/cag.12463

**Published:** 2018-04-25

**Authors:** Alina Ristea, Martin A. Andresen, Michael Leitner

**Affiliations:** ^1^ Doctoral College GIScience, Department of Geoinformatics‐Z_GIS University of Salzburg; ^2^ Institute for Canadian Urban Research Studies School of Criminology Simon Fraser University; ^3^ Department of Geography and Anthropology Louisiana State University

**Keywords:** spatial crime analysis, Twitter, hockey, geographically weighted regression, analyse spatiale de la criminalité, Twitter, hockey, régression pondérée géographiquement

## Abstract

The use of social media data for the spatial analysis of crime patterns during social events has proven to be instructive. This study analyzes the geography of crime considering hockey game days, criminal behaviour, and Twitter activity. Specifically, we consider the relationship between geolocated crime‐related Twitter activity and crime. We analyze six property crime types that are aggregated to the dissemination area base unit in Vancouver, for two hockey seasons through a game and non‐game temporal resolution. Using the same method, geolocated Twitter messages and environmental variables are aggregated to dissemination areas. We employ spatial clustering, dictionary‐based mining for tweets, spatial autocorrelation, and global and local regression models (spatial lag and geographically weighted regression). Findings show an important influence of Twitter data for theft‐from‐vehicle and mischief, mostly on hockey game days. Relationships from the geographically weighted regression models indicate that tweets are a valuable independent variable that can be used in explaining and understanding crime patterns.

## Utiliser des gazouillis pour comprendre les changements dans la distribution spatiale de la criminalité lors des parties de hockey à Vancouver

L'utilisation des données des médias sociaux pour l'analyse spatiale des tendances de la criminalité durant des activités sociales s'est avérée très instructive. Cette étude analyse la géographie de la criminalité compte tenu des journées où il y a une partie de hockey, le comportement criminel et l'activité sur Twitter. Plus précisément, nous examinons les relations entre la criminalité et l'activité sur Twitter reliée à la criminalité géolocalisée. Nous analysons six types de crimes contre les biens qui sont agrégés par aire de diffusion à Vancouver pour deux saisons de hockey au moyen d'une résolution temporelle avec et sans partie. Utilisant la même méthode, les messages géolocalisés sur Twitter et les variables environnementales sont agrégés aux aires de diffusion. Nous utilisons le regroupement spatial, l'extraction basée sur le dictionnaire pour les gazouillis, l'autocorrélation spatiale ainsi que les modèles locaux et globaux de régression (décalage spatial et régression pondérée géographiquement). Les conclusions indiquent une influence importante des données de Twitter pour les méfaits et les vols dans les véhicules, principalement lors des journées où il y a une partie de hockey. Les relations des modèles de régression pondérée géographiquement indiquent que les gazouillis sont une variable indépendante utile qui peut être utilisée pour expliquer et comprendre les tendances de la criminalité.

## Introduction

Spatial patterning of crime research most often involves routine activity theory, social disorganization theory, and the geometry of crime (Andresen 2006, 2011). Research invoking routine activity theory to explain spatial crime patterns has found that particular routine activities (spending time away from the relatively protective environment of the home) and places that attract large volumes of people on a routine basis experience larger volumes of crime (Kennedy and Forde 1990; Andresen 2011).

However, non‐routine activities may be able to explain short‐term changes in those spatial patterns. Such non‐routine activities must be significant enough to change the spatial distribution of motivated offenders, suitable targets, and capable guardians, albeit for a short period of time. Investigations into these possible changes need to have crime data, and other explanatory data, that are spatially and temporally available at a relatively fine resolution.

The geometry of crime (Brantingham and Brantingham 1981) focuses on the possible intersection between the activity spaces of an offender and a victim. This intersection could occur at different activity nodes (e.g., pubs, fast food restaurants, alcohol outlets) or along their pathways. These types of nodes sometimes act as crime generators and crime attractors (Brantingham and Brantingham 1995). Several researchers have noted that stadiums can be both a crime generator and attractor for distinct place‐ and time‐specific events (Ratcliffe 2004; Kurland et al. 2014; Brantingham et al. 2017).

In this paper we consider one such non‐routine activity that emerges often enough, but not with the typical periodicity of a crime generator or attractor: spatial crime pattern changes resulting from a sporting event, namely hockey. Because of the volume of people who attend hockey games (almost 20,000 in the arena), plus all of the other individuals who are out watching the game in sports bars and other related drinking establishments, it is possible that some crime types may experience a change in their spatial patterns when these games occur.

There is a growing literature that investigates the impact on crime from sporting events (football in Europe, for example), as well as a growing literature that shows how peoples’ behaviour on social media changes during events. However, there is limited research that investigates the relationship, if present, between sporting events, social media activity, and criminal events. Focusing on professional hockey in Vancouver, British Columbia, we undertook such an analysis. We separated the spatial distribution of crime into three categories: home‐based game days, away‐based game days, and non‐game days. We examined the associated Twitter activity on those days, controlling for a number of other factors. We anticipated that home‐based game days should have the greatest change in crime patterns because people will be at the arena and at drinking establishments, followed by away‐based game days, and non‐game days. Overall, we found that criminal events do increase during home‐based game days, but the effect varies by crime type.

## Sporting events, crime, and social media

Sporting events attract large volumes of people at specific locations (e.g., arenas) and general areas that contain alcohol outlets. These sporting events have positive impacts on society through social and economic aspects: income, employment, tourism, and a betting market (Hopkins and Treadwell 2014; Kain and Logan 2014; Kurland et al. 2014). However, there can be negative aspects to these events including fan behaviour (Montolio and Planells 2015), hooliganism (Kurland et al. 2014), and criminal behaviour (Breetzke and Cohn 2013).

Recent research in this area has focussed on football games in Europe and the spread of hooliganism (Planells‐Struse 2015). This research has found the following: distance from the sporting event predicts where thefts (900–1100 metres) and assaults (600–700 metres) may occur (Montolio and Planells 2016); violent and property crime increases within the immediate vicinity of events, with property crime increasing in the half‐mile to one‐mile range (Billings and Depken II 2012); drunk and disorderly behaviour increases within the half‐mile to one‐mile range (Breetzke and Cohn 2013); and criminal damage, theft, and violence increases within a 3‐kilometre range (Kurland et al. 2014). Temporally, research has found that these increases in criminal activity are within particular time ranges before and after the sporting events: seven hours prior to and after Football Club Barcelona home matches (Montolio and Planells 2016); six hours prior to and after home and away matches for nine stadia in London (seven neighbourhoods) (Marie 2016); six hours prior to and after for basketball matches and robberies in Memphis, Tennessee (Yu et al. 2016); three hours prior to and two hours after for the Super Bowl in Chicago (Laqueur and Copus 2014); and four hours prior to and after matches for Hillsborough football ground in the United Kingdom (Kurland 2014). As such, both space and time must be considered when investigating changes in the patterning of criminal events related to sporting events.

Social media research has shown that organized events, such as sporting events, are related to spikes in social media volume (Cheng and Wicks 2014). Moreover, Twitter data have been used to detect specific moments during football matches—such as goals, red cards, or penalties—using semantic or “sentiment” analysis (Kampakis and Adamides 2014). These data can also be used to predict the outcome of football matches by combining historical outcomes and semantic analysis of tweets in machine‐learning models and regression analysis (Kampakis and Adamides 2014). As such, these studies show the possibility of combining historical crime data and semantic analysis of tweets to investigate predictive models for crime occurrences for the hockey games timeframe.

## Data and methods

The city of Vancouver is the primary city within Metro Vancouver, the third largest metropolitan area in Canada. The city of Vancouver had a population of approximately 630,000 in 2016 (Statistics Canada 2017a), making it the eighth largest city in Canada and the fourth largest city in western Canada—with 2.4 million people in the Metro Vancouver area (Statistics Canada 2017b). The large population area of Metro Vancouver, encompassing areas just outside and surrounding the city, serves as a population base for spectators who are drawn into the city during hockey games.

Ice hockey is the national sport in Canada (Marsh 2013), a country considered to be its birthplace. Hockey riots have occurred twice in Vancouver: the first was in 1994 after the Vancouver Canucks (Vancouver's National Hockey League team) lost the Stanley Cup to the New York Rangers in Game 7 at Madison Square Gardens in New York; the second was in 2011 when the team lost the same cup in the final with Boston Bruins, while playing in Vancouver (Schneider and Trottier 2012). While the Vancouver Police Department successfully controlled large crowds during the 2010 Winter Olympics, in 2011, hockey fans managed to destroy cars, shops, and start fires on the streets, leading to 140 non‐fatal injuries and an estimated C$5 million in property damage (Bailey 2011).

### Data

The crime data for Vancouver are police incident data retrieved from the Vancouver Open Data Catalogue (City of Vancouver 2017). These data contain information for location, date, and time for six property crime types since 2003: residential burglary, commercial burglary, mischief, theft‐from‐vehicle, theft‐of‐vehicle, and other theft. Other theft includes theft of property that does not include violence and is not captured under the other property crime classifications. Mischief is willful malicious destruction, damage, or defacement of property. We analyzed disaggregated crime types because of their known differences in spatial patterns (Andresen and Linning 2012) and included crime data from 2014–2016 for the time periods corresponding to the hockey season.

Previous literature shows the importance of liquor‐related locations, restaurants, amenities, and transportation hubs for spatiotemporal crime occurrences (Kinney et al. 2008; Bernasco and Block 2011; Grubesic and Pridemore 2011; Groff and Lockwood 2014). In order to control for these effects, we downloaded the following variables from the Vancouver Open Data Catalogue: population, public roads, parks, street parking, street light poles, rapid transit stations, traffic signals, public washrooms, and liquor businesses. Our study area included 995 dissemination areas (DA).

Geolocated tweets were obtained using the Twitter Streaming Application for 2014–2016 (Twitter, Inc. 2017), which offers the possibility to download tweets free of charge applying different filters and accessing tweet content and attributes such as user name, user location, and message time. Although Twitter data are commonly used in research, several limitations may arise related to the availability of geolocation and socio‐demographics (Morstatter et al. 2013; Zhang et al. 2016; Resch et al. 2017). See Steiger et al. (2015) and Sui and Goodchild (2011) for discussions of these limitations.

Time and date information for hockey games were collected from online databases for the Vancouver Canucks for the 2014–2015 and 2015–2016 seasons that occur approximately between October and April each year (Hockey Reference 2017). We considered two types of game days: home and away; comparison days (mostly represented by the second day after a match, later if subsequent games are close in time) were also separated for home and away games. There is no accepted timeframe defined for criminogenic effects before and after sport events, so for this case study we defined a timeframe of four hours before the start and four hours after the start of the game. The crime types and the geolocated tweets are temporally (hourly) pre‐processed for the eight‐hour timeframe: 4,316 crimes home games, 4,100 crimes comparison home games, 4,012 crimes away games, 4,146 crimes comparison away games; 60,339 tweets home games, 56,553 tweets comparison home games, 60,374 tweets away games, 59,814 tweets comparison away games. The crimes, tweets, and chosen environmental variables were aggregated to dissemination areas.

We also undertook a semantic approach for the Twitter data, extracting crime‐related tweets (crime‐tweets). The dictionary was compiled by the authors, including violent words from online dictionaries (Vocabulary University 2017) and the names of all crime types according to Canada's *Criminal Code*. This is a naïve approach that considers pre‐defined offensive and violent seed words—e.g., “murder,” “attack,” “enemy,” and “gang.” This action was applied for the four Twitter datasets. After this step, we had the following subsets: 2,977 crime‐tweets home games, 2,760 crime‐tweets comparison home games, 2,975 crime‐tweets away games, 2,843 crime‐tweets comparison away games. For example, a tweet such as “Mounties have arrested suspect in pair of poppy donation box thefts in Nanaimo” is considered a crime‐tweet because it includes a crime type in the text.

### Methods

We considered three different steps for analyzing our data. First, in order to identify spatial clustering, we considered monthly temporal representation of the data as well as Moran's *I*. Second, spatial lag (SL) global regressions were used to determine significant explanatory variables for crime occurrences, while accounting for any spatial autocorrelation in the data. Because of the number of regressions run and the complications that may emerge from multiple hypothesis‐testing, the p‐values were corrected using the Benjamini and Hochberg method (Benjamini and Hochberg 1995). Regression diagnostics were checked for all models. These diagnostics indicated no problems with multicollinearity and showed that the SL models had improved goodness‐of‐fit over ordinary least squares models based on log‐likelihood and Akaike information criterion (AIC). However, it is important to note that a global model offers only an overview, or spatial average, of the explanatory variables in the study area, which can mask local spatial relationships (Brunsdon et al. 1996, 1999; Fotheringham et al. 1997, 1998).

The third step was to run a geographically weighted regression (GWR) in order to identify any spatial influence of the explanatory variables over crime occurrences, resulting in four models for each crime type (a total of 24 models) and another four models for aggregated crime types. GWR is a regression model adapted for a local spatial analysis perspective (Brunsdon et al. 1996, 1999; Fotheringham et al. 1997, 1998). GWR allows for the identification of parameter estimates varying across space. In the current context, measures of Twitter activity may be statistically significant around the sporting arena and not elsewhere.

## Results

**Figure 1:**
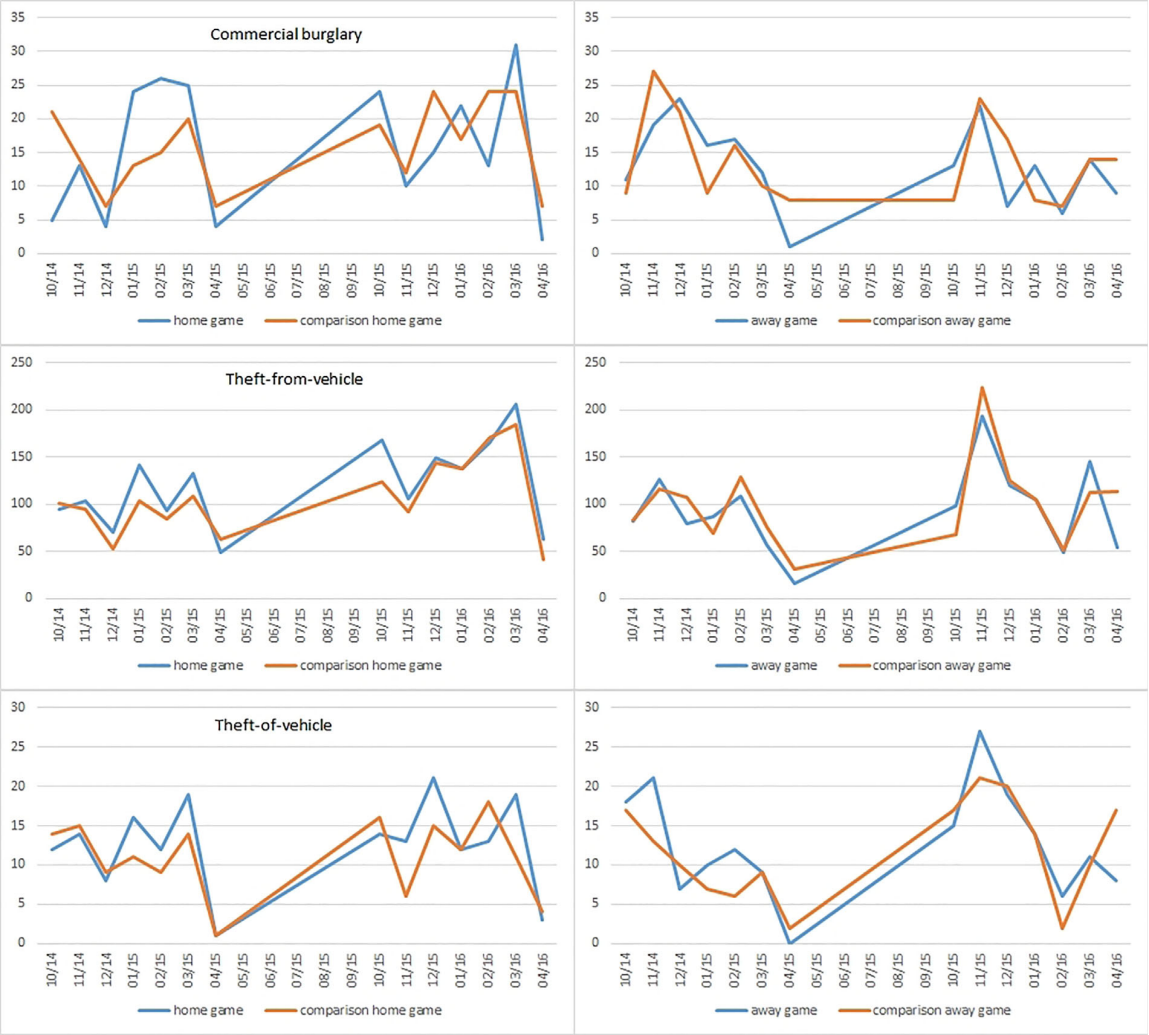
Temporal distribution of crime occurrences.

Figure 1 shows there are increased differences for home‐game days relative to comparison home‐game days for commercial burglary (January and March 2015; March 2016), theft‐from‐vehicle (October 2015), and theft‐of‐vehicle (January and March 2015; March 2016). Moreover, the graphical pattern shows increases for the away‐game days compared with comparison away‐game days for theft‐from‐vehicle (March 2016) and theft‐of‐vehicle (November 2014; February 2015; November 2015). These differences must be interpreted with caution, however, because these may occur simply from random variation in the data.

With regard to hotspot clustering, we found different patterns for game days and comparison days. However, it should be noted that not all crime types occur with greater frequency in the immediate vicinity of Rogers Arena (the Canucks’ home stadium) on game days, but there is an increase in the degree of clustering. Aggregated crime types show an increased volume in Downtown and around the stadium for home games, and more crime in the Mount Pleasant and Fairview areas away from the stadium, during comparison away games. Turning to the disaggregated crime types, theft‐from‐vehicle, other theft, and commercial burglary show increased concentrations around Rogers Arena and Downtown for home games. Interestingly, mischief comparison days had a higher density than mischief home games in Downtown North, with Downtown having more crimes during home games (39 compared with 25). For residential burglary, no relationships were found between crime densities around the stadium; however, patterns were changing across the city in the four temporal frames.

Considering the bivariate spatial autocorrelation between crime with geolocated tweets and crime‐tweets, where tweets were considered a lag in the models, the Moran's I index values for aggregated crimes and geolocated tweets was 0.26; for comparison home games, 0.26; for away games, 0.26; and for comparison away games, 0.24. In the same order, replacing tweets with the crime‐tweets, the index values increased to 0.29, 0.28, 0.27, and 0.25, respectively (all of them significant, p < 0.01). In the current context, crime‐tweets are more correlated in space with aggregated crimes and also with disaggregated crimes during home games and away games.

### SL results

The SL models were run for each timeframe (home games, comparison home games, away games, comparison away games) for the aggregated crimes and the disaggregated crimes, generating a total of 56 models (28 models included geolocated tweets and environmental variables as explanatory variables; the other 28 included crime‐tweets and environmental variables). The coefficients for population information from the 2011 Census, parks, disabled parking, and washrooms were statistically significant the least often in these models. The coefficients for crime‐tweets, light poles, liquor stores, and public roads were statistically significant most frequently in the 56 models. For the aggregated crime types, there were variables that were significant for all timeframes: tweets, crime‐tweets, light poles, traffic signals, liquor stores, and public roads (Table 1).

**Table 1:** SL coefficients (black: significant; grey: not significant)

Dependent variable		R^2^	Tweets	Crime‐tweets	Pop. 2011	Parks	Disable parking	Street parking	Motor parking	Light poles	Transit stations	Traffic signs	Wash‐ rooms	Liquor stores	Public roads
aggregated crimes	home games	0.797	0.017		−0.001	−0.988	2.695	0.033	−0.559	0.074	6.897	1.844	−0.419	1.365	−0.119
		0.869		0.639	0.000	−0.820	2.008	−0.003	−0.773	0.042	1.550	1.261	−1.034	1.042	−0.061
	away games	0.780	0.015		−0.001	−1.211	1.434	0.047	−0.613	0.064	9.756	2.164	0.647	1.418	−0.102
		0.838		0.532	0.000	−1.163	0.957	0.025	−0.089	0.032	3.767	1.644	0.298	1.206	−0.056
	comparison home	0.783	0.013		−0.002	−0.978	1.959	0.050	0.046	0.068	9.675	2.086	0.747	1.391	−0.093
		0.841		0.624	0.000	−0.944	1.205	0.005	−1.224	0.024	6.917	1.783	0.338	1.232	−0.059
	comparison away	0.773	0.016		−0.001	−0.626	2.287	0.058	−0.313	0.075	9.065	2.328	0.734	1.097	−0.122
		0.831		0.595	0.000	−0.502	1.776	0.033	−0.903	0.030	4.146	1.949	0.379	0.830	−0.070
home‐game days	theft‐from‐vehicle	0.743	0.008		0.000	−0.411	0.247	0.005	−0.545	0.024	1.950	0.541	−0.251	0.305	−0.051
		0.796		0.243	0.001	−0.365	0.003	−0.006	−0.535	0.012	0.421	0.343	−0.394	0.236	−0.032
	theft‐of‐vehicle	0.131	0.000		0.000	−0.024	0.026	0.001	0.050	0.002	−0.171	0.000	−0.033	−0.012	−0.001
		0.131		0.002	0.000	−0.023	0.023	0.001	0.047	0.002	−0.199	−0.002	−0.037	−0.014	−0.001
	mischief	0.592	0.001		0.000	−0.069	0.601	0.000	−0.241	0.010	0.720	0.257	−0.010	0.181	−0.018
		0.657		0.060	0.000	−0.050	0.536	−0.004	−0.280	0.007	0.125	0.197	−0.087	0.140	−0.012
	other theft	0.755	0.006		−0.001	−0.510	1.637	0.025	0.278	0.029	5.006	0.873	−0.242	0.704	−0.036
		0.829		0.290	0.000	−0.420	1.303	0.008	0.117	0.014	2.247	0.596	−0.578	0.525	−0.009
	commercial burglary	0.467	0.000		0.000	−0.027	0.099	0.002	0.062	0.018	0.073	0.058	0.087	0.007	−0.003
		0.468		0.004	0.000	−0.026	0.095	0.002	0.061	0.003	0.045	0.055	0.084	0.006	−0.002
	residential burglary	0.075	0.000		0.000	−0.008	−0.087	0.000	−0.081	0.003	−0.185	−0.003	−0.040	0.011	0.003
		0.074		−0.001	0.000	−0.008	−0.086	0.000	−0.085	0.003	−0.199	−0.003	−0.044	0.010	0.003
away‐game days	theft‐from‐vehicle	0.651	0.006		0.000	−0.549	0.453	0.014	−0.883	0.015	3.922	0.769	0.485	0.416	−0.040
		0.739		0.207	0.000	−0.524	0.249	0.006	−1.013	0.002	1.583	0.567	0.337	0.321	−0.022
	theft‐of‐vehicle	0.175	0.000		0.000	0.015	−0.001	0.001	0.086	0.001	−0.158	0.037	−0.013	−0.012	0.002
		0.178		0.003	0.000	0.017	−0.012	0.001	0.075	0.000	−0.223	0.034	−0.021	−0.019	0.002
	mischief	0.649	0.001		0.000	0.021	−0.054	0.001	−0.021	0.009	0.390	0.198	0.007	0.144	−0.013
		0.670		0.027	0.000	0.022	−0.078	0.000	−0.036	0.007	0.088	0.172	−0.012	0.131	−0.011
	other theft	0.757	0.008		−0.001	−0.531	1.121	0.030	0.287	0.037	4.971	1.021	−0.074	0.566	−0.044
		0.808		0.271	−0.001	−0.505	0.879	0.019	0.179	0.021	2.002	0.769	−0.226	0.482	−0.022
	commercial burglary	0.375	0.000		0.000	−0.029	−0.115	0.000	0.088	0.000	0.494	0.058	0.058	0.078	−0.002
		0.377		−0.005	0.000	−0.030	−0.110	0.000	0.089	0.000	0.544	0.062	0.060	0.079	−0.002
	residential burglary	0.115	0.000		0.000	−0.106	−0.093	−0.001	−0.029	0.002	−0.106	−0.015	0.168	0.039	0.006
		0.118		−0.005	0.000	−0.108	−0.083	−0.001	−0.021	0.002	−0.038	−0.011	0.174	0.043	0.005
comparison home‐game days	theft‐from‐vehicle	0.717	0.004		0.000	−0.370	−0.003	0.020	−0.235	0.020	2.556	0.556	0.416	0.339	−0.030
		0.764		0.178	0.000	−0.365	−0.201	0.008	−0.559	0.008	1.896	0.474	0.326	0.318	−0.021
	theft‐of‐vehicle	0.200	0.000		0.000	−0.063	−0.021	0.001	0.074	0.002	0.062	0.025	0.031	−0.001	−0.001
		0.200		−0.001	0.000	−0.063	−0.021	0.001	0.074	0.002	0.061	0.026	0.030	−0.003	−0.001
	mischief	0.614	0.001		0.000	−0.095	0.129	0.003	−0.117	0.005	1.024	0.221	0.191	0.116	−0.011
		0.624		0.027	0.000	−0.097	0.107	0.002	−0.159	0.003	0.947	0.208	0.182	0.116	−0.010
	other theft	0.728	0.007		−0.001	−0.506	1.862	0.020	0.326	0.037	6.259	1.210	0.029	0.691	−0.048
		0.805		0.388	0.000	−0.473	1.349	−0.008	−0.511	0.009	4.410	1.027	−0.252	0.569	−0.027
	commercial burglary	0.527	0.000		0.000	−0.004	−0.094	0.005	0.065	0.002	0.335	0.066	0.064	0.046	−0.002
		0.526		−0.002	0.000	−0.003	−0.096	0.005	0.063	0.002	0.326	0.067	0.060	0.042	−0.002
	residential burglary	0.079	0.000		0.000	−0.021	−0.056	−0.056	0.001	−0.071	0.000	−0.001	−0.005	0.009	0.010
		0.079		0.000	0.000	−0.021	−0.056	0.001	−0.070	0.000	0.001	−0.032	−0.004	0.009	0.010
comparison away‐game days	theft‐from‐vehicle	0.713	0.005		0.000	−0.499	0.394	0.020	−0.685	0.023	3.550	0.775	0.847	0.321	−0.051
		0.771		0.187	0.000	−0.459	0.232	0.012	−0.870	0.009	2.054	0.657	0.739	0.240	−0.035
	theft‐of‐vehicle	0.086	0.000		0.000	−0.005	0.011	−0.002	−0.001	0.001	−0.274	0.044	−0.013	0.021	−0.002
		0.088		0.002	0.000	−0.004	0.004	−0.002	−0.009	0.001	−0.309	0.042	−0.017	0.017	−0.001
	mischief	0.637	0.001		0.000	0.086	−0.024	0.005	0.088	0.009	0.445	0.133	0.017	0.079	−0.010
		0.663		0.036	0.000	0.093	−0.052	0.004	0.061	0.006	0.180	0.111	0.001	0.068	−0.007
	other theft	0.716	0.009		−0.001	−0.391	1.964	0.028	0.439	0.040	4.374	1.163	−0.209	0.493	−0.047
		0.784		0.350	0.000	−0.305	1.625	0.013	0.069	0.013	1.394	0.952	−0.425	0.329	−0.017
	commercial burglary	0.594	0.000		0.000	−0.053	−0.056	0.004	−0.013	0.002	0.694	0.071	0.050	0.054	−0.004
		0.591		0.005	0.000	−0.054	−0.052	0.003	−0.004	0.002	0.696	0.069	0.056	0.060	−0.003
	residential burglary	0.045	0.000		0.000	0.109	−0.061	−0.001	−0.023	−0.023	0.000	−0.143	−0.003	0.014	0.002
		0.045		0.000	0.000	0.110	−0.062	−0.001	−0.025	0.000	−0.150	−0.003	−0.041	0.013	0.002

Interestingly, crime‐tweets were statistically significant in all the cases that geolocated tweets were significant, with a positive parameter during away games for commercial burglary. Moreover, the magnitudes of the estimated parameters were considerably greater for crime‐tweets, comparing them for: crime on home‐game days (0.02 for tweets, 0.64 for crime‐tweets); crime on away‐game days (0.02 and 0.53); crime on comparison home days (0.01 and 0.62); and crime on comparison away games (0.02 and 0.60). Also, considering theft‐from‐vehicle, mischief, and other theft, there was a large variation between tweets and crime‐tweets coefficients. This is interesting because the presence of tweets can be used as a measure of the ambient population that is at‐risk for criminal victimization (Malleson and Andresen 2015, 2016; Kounadi et al. 2017), and is positively related to criminal activity; however, the presence of crime‐tweets was a much stronger predictor of changes in the volume of crime. It is worth noting that the distributions for tweets and residential population differ in the study area, thus both explanatory variables were used for crime regression models so that population at‐risk for crime could be represented by a calculated variable from these data. We did not find a significant difference between game days and their comparison days. In some cases, crime‐tweets had a greater magnitude parameter, in other cases they did not. As such, for the global results, there did not appear to be an impact from the sporting‐event days on regression results.

### GWR results

As discussed above in the context of global models, GWR highlights the importance of understanding space and place for criminal activity. A total of 56 GWR models were run, using the same dependent and explanatory variables as the SL models. GWR was utilized in an exploratory manner to examine the spatial performance of the local models and determine whether these explicitly spatial models were more appropriate than the global models. However, it is important to mention that by changing the model parameters, i.e., excluding the explanatory variables which were not statistically significant in the SL models, the GWR spatial pattern for tweets and crime‐tweets was similar.

The GWR global output for each model includes bandwidth, effective number, sigma, AIC, R^2^, and adjusted R^2^. It is interesting to note that the R^2^ and adjusted R^2^ values were all quite high for the aggregated crime types, with adjusted R^2^ values ranging from 0.88 to 0.91. Theft‐from‐vehicle and other theft crime types also performed very well, with adjusted R^2^ values ranging from 0.81 to 0.88 for the four temporal frames, followed by mischief with adjusted R^2^ values ranging from 0.67 to 0.73. The remaining crime types, however, had low adjusted R^2^ values.

In addition to the global GWR statistics, each model includes local statistics for each DA in the current context: local R^2^, predicted values, coefficient for each explanatory variable, residuals, standard error, standard error coefficient for each explanatory variable, and standard residuals. Given the study's principal purpose of examining the relationship between Twitter data and crime with a spatial lens, the local R^2^ values were mapped to reveal if there were differences across study zones in the model's ability to explain variation.

Mapping the crime‐tweets coefficient for R^2^ using the aggregated crime types results showed a low influence in the southern portion of Vancouver, relatively distant from the sporting arena and much of the alcohol establishment district. The influence of crime‐tweets increased moving north in Vancouver, with higher values in the Downtown, Stanley Park, and English Bay areas. For home games, the values ranged between −0.01 to 0.80, which indicates a higher influence of crime‐tweets than for comparison home games, which ranged from −0.26 to 0.76. The opposite pattern emerged for away‐game days: comparison away games had a maximum value of 0.94, whereas away games had a maximum value of 0.72. It is important to note that in all cases, geographic proximity to the sporting arena and the alcohol district led to an increase in R^2^ values.

Twitter activity had a low local influence on four of the six analyzed crime types. As discussed above, residential burglary and theft‐of‐vehicle models were very weak, with adjusted R^2^ values ranging from 0.1 to 0.2. Commercial burglary models had very low‐magnitude coefficients for the crime‐tweets, between −0.03 and 0.05. The spatial pattern of the coefficients for these three crime types—residential burglary, theft‐of‐vehicle, and commercial burglary—during home games is quite different from the other crime types, not following the expected pattern, which is hierarchical from north to south, low to high, with the highest values being close to the sporting arena. Generally speaking for these crime types, there is very weak, or no, influence close to the sporting arena or the Downtown area, so the present analysis will not focus on these crime types. An interesting result did emerge for the other theft crime‐tweets coefficient. In this case, the explanatory variable had an increased value for comparison days rather than game days. Also, the difference between the minimum and maximum values was higher than for other variables: home games −0.08 to 0.42; comparison home games −0.13 to 0.52; away games 0.01 to 0.37; and comparison away games −0.02 to 0.62. Consequently, these results showed that in some instances, crime‐tweets may be an important variable to be used in analyses that are not focusing on specific events.

**Figure 2:**
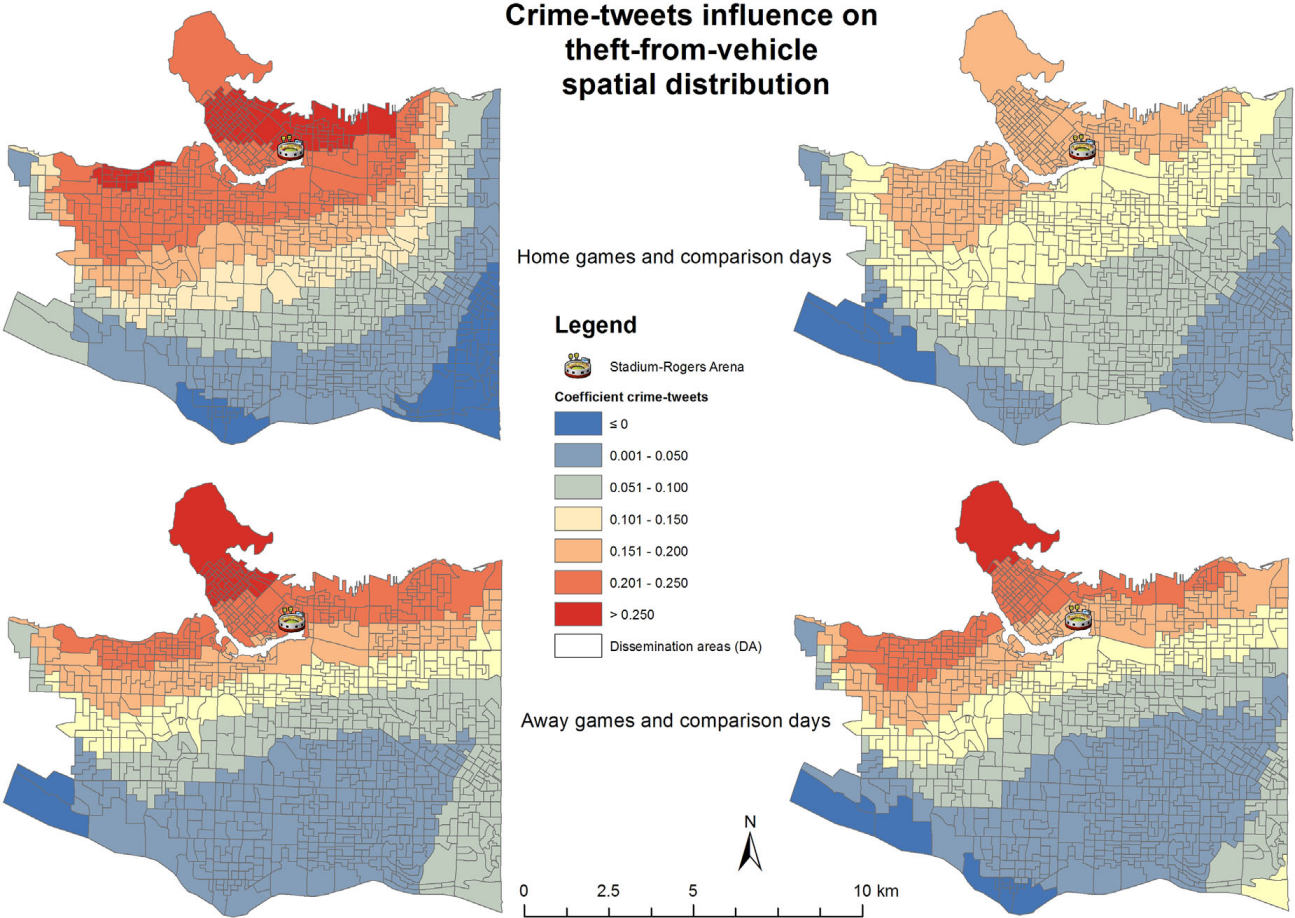
Crime‐tweets coefficient on theft from vehicle GWR models.

**Figure 3:**
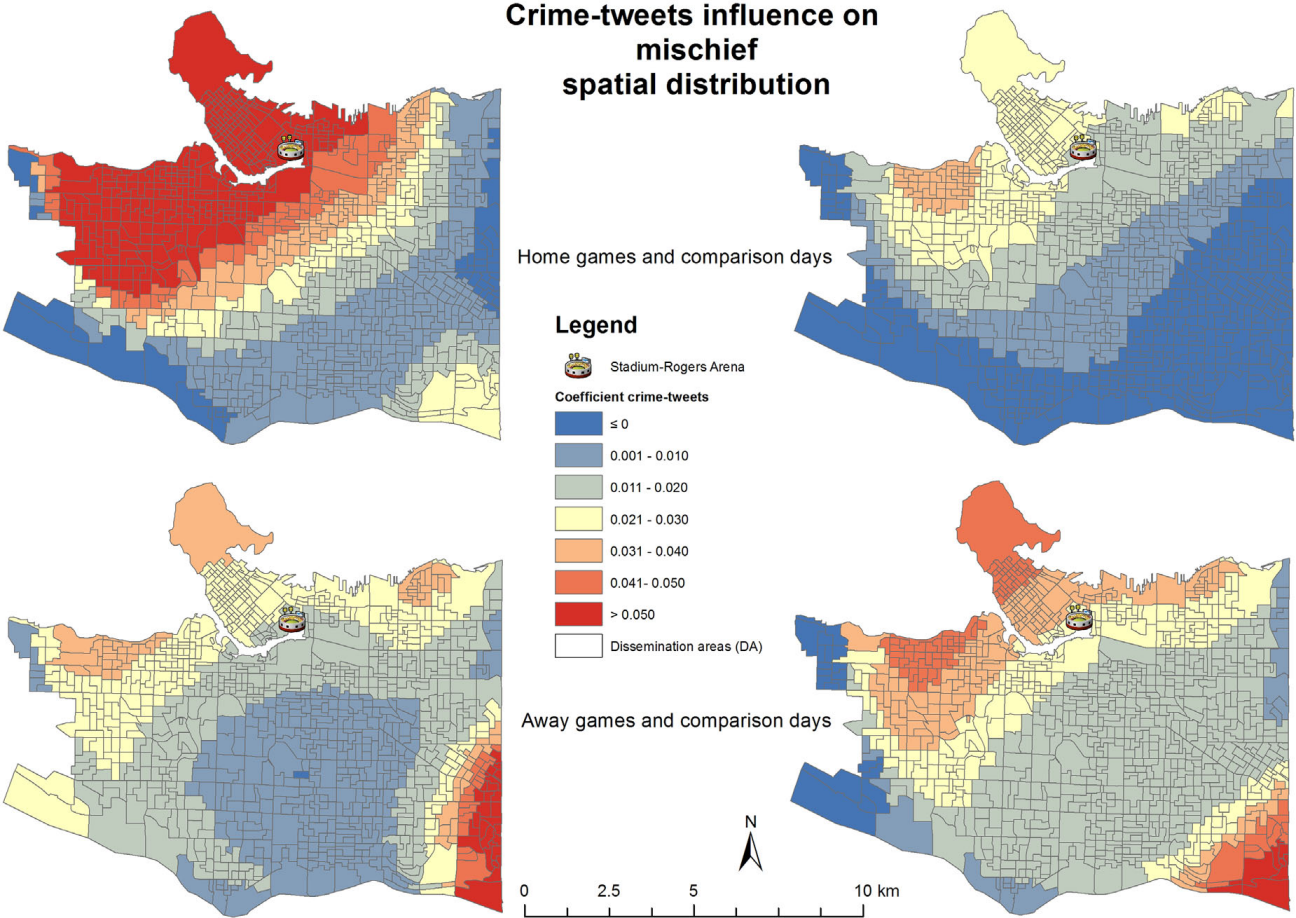
Crime‐tweets coefficient on mischief GWR models.

Theft‐from‐vehicle and mischief showed the importance of using Twitter data as an explanatory variable. In the upper portion of Figure 2, the neighbourhoods of Stanley Park, Downtown, East Downtown, Yaletown, False Creek, Point Grey, Kitsilano, and Mount Pleasant East all show a moderate impact of crime‐tweets (0.23–0.26) for home games. The same pattern is present for comparison home games, but at a lower magnitude. In the lower portion of Figure 2, the pattern is similar for away games and comparison away games; however, for away games the highest values (0.23–0.30) cover part of Downtown and Stanley Park, while for comparison away games the influence is not so strong in the Downtown DAs. Looking at mischief in Figure 3, it is worth noting that the model coefficients range between −0.01 to 0.08, so the influence was not as strong as it was for theft‐from‐vehicle. However, crime‐tweets used during home games reveal a different intensity for mischief. This is not the case for away games when the spatial pattern is similar, but comparison away games are more intense in Downtown, Stanley Park, and in the western part of the city.

As with the SL models, liquor stores and traffic signs were the explanatory variables with the largest magnitude coefficients. As such, after finding the influence of crime‐tweets for the theft‐from‐vehicle and mischief crime types, we also tested these coefficients on a local scale. In the case of theft‐from‐vehicle, liquor establishments had a higher influence in the central part of the city and were present for all temporal frames; moreover, this spatial pattern is more pronounced for home games in the city centre (maximum 0.52, compared with 0.46 for comparison home games). Returning to Table 1, SL values for liquor influence (home games 0.31/0.24 and comparison home games 0.34/0.32) show the importance of a local model. Regarding the traffic signs variable, the pattern showed a greater influence in the Downtown area and decreases in a circular pattern as one moves away from that area: home games −0.12 to 1.11; comparison home games −0.008 to 1.14; away games −0.18 to 1.30; comparison away games −.19 to 1.52.

For mischief, liquor stores had similar patterns as in theft‐from‐vehicle models, with higher influence in the central part of the city that decreased as one moved away from the centre of the city. The influence was stronger for game days (home games −0.13 to 0.29 and away games −0.13 to 0.34) compared to comparison days (comparison home games −0.05 to 0.17 and comparison away games −0.30 to 0.12). Again, because of the spatial variation in these coefficients, SL values might be misleading in this type of study—with values of 0.18/0.14 for home games; 0.14/0.13 for away games; 0.16/0.16 for comparison home games; and 0.08/0.07 for comparison away games. It is notable the influence of traffic signs was also stronger for game days; home games −0.07 to 0.51; away games −0.11 to 0.35; comparison home games −0.02 to 0.45; comparison away games −0.07 to 0.27. The spatial patterns were similar with theft‐from‐vehicle.

## Discussion

This study provides insight about the utility of illustrating spatial relationships between criminal activity, Twitter activity, and other socio‐demographic and economic variables, in the context of sporting events. Specifically, we are able to show that crime‐related Twitter activity can be an explanatory variable for criminal activity, when considered together with environmental factors and other population data. We applied our research design to two professional hockey game seasons in Vancouver (2014–2015 and 2015–2016), using a game and comparison game day approach, considering home and away games in a timeframe of four hours prior to and after each game. The areal unit for the study is represented by the 995 DAs. Six crime types were analyzed: commercial burglary, residential burglary, mischief, other theft, theft‐from‐vehicle, and theft‐of‐vehicle. Geolocated tweets were selected for the same timeframe as crimes. Also, crime‐tweets are considered as a geolocated subset and contain tweets using violent and crime‐related words. The relationships between a set of explanatory variables and the different crime types are allowed to vary, thus the analysis is provided with flexibility in choosing the variables with possible relationships with social disorganization theory, the geometry of crime, and routine activity theory in the Vancouver DAs. In addition to crime and tweets, we included additional information in our spatial models, such as: population data from Census 2011, parks, public roads, street parking, disability parking, motor vehicles parking, street light poles, rapid transit stations, traffic signals, pubic washrooms, and liquor stores.

Aggregating the count of crimes per month for the two hockey seasons, we found changes in the monthly temporal patterns for commercial burglary, theft‐from‐vehicle, and theft‐of‐vehicle crime types when comparing home and away games with comparison days. However, commercial burglary and theft‐of‐vehicle values did not have a high increase in crime occurrences, so we consider only theft‐from‐vehicle monthly patterns to be related with hockey games. This supports the work of Kirk (2008), which found that assaults and theft‐from‐vehicle rose during study period of hockey games. We also found that spatial concentrations of crime had different patterns for game and comparison days. Aggregated crimes, theft‐from‐vehicle, other theft, and commercial burglary showed increased concentrations around Rogers Arena and the Downtown area for home games. This was to be expected, considering the increase in the number of cars around the area where the hockey games occur, and the increase in the number of people in the area because of the game attendance (Kirk 2008). Commercial burglary, however, had an unexpectedly higher density, although this finding might not be large in magnitude because the difference in density was not that high (e.g., in one of the Downtown DAs it rose from five to seven crimes). Bivariate spatial autocorrelations show the importance of considering a full dataset of geolocated tweets or using a subset of crime‐tweets.

The SL results showed the importance of the geolocated tweets and crime‐tweets and underscored the need to use an appropriate at‐risk population when examining different crime types (Malleson and Andresen 2016). The ambient population, which is represented by the tweets, might not be always sustainable as the population‐at‐risk, particularly for crime types where the presence of population in space is seen to represent guardianship and could affect the possible offender. We noticed in our results that theft‐of‐vehicle, commercial burglary, and residential burglary show low or no significance in the tweets. This supports the bivariate spatial autocorrelation results where residential burglary and theft‐of‐vehicle had insignificant values. However, because the residential population is also statistically insignificant, this raises the question of which population is likely best to use to reduce errors when analyzing theft‐of‐vehicle, commercial burglary, and residential burglary crime types?

Another important result from the SL models is that the crime‐tweets subset variable was a better explanatory variable than all geolocated tweets analyzed together with the additional variables. This is notable because it shows that not all the people's locations are a proxy for where crimes may occur. However, using of tweets, or a subset of them, depends on the purpose of analyses. For this study, investigating how social media information might interfere with crimes for specific events, the subset was important. For a more general purpose, excluding the presence of an event, it might be more useful to consider a full geolocated dataset.

Overall, the most notable result from the GWR models was the consistency with theoretical expectations. Firstly, for the aggregated crime types, the models for hockey games played at home or away showed increase strength of crime‐tweets as a possible explanatory variable, compared to the comparison days with no games. As a spectator event, hockey game days attract a large volume of people and are associated with a higher number of crime‐tweets. This finding indicates that the full set of geolocated tweets and/or subsets of Twitter data may be important explanatory variables for overall crimes occurring in the city during the event temporal resolution. Specifically, the GWR models showed that two of the six crime types had a stronger connection with crime‐tweets, namely theft‐from‐vehicle and mischief.

According to the theoretical background of routine activity theory (Cohen and Felson 1979), changes in the perceived space for usual activities may increase crime occurrences through an exposure to motivated offenders, higher target attractiveness, or the lack of guardianship. These discussions are relevant here for theft‐from‐vehicle during home games because people may offer suitable opportunities for offenders, such as parking in low surveillance places or leaving goods in their vehicle, which can serve as a crime generator (Brantingham and Brantingham 1981). Also, other risk factors present in the area of the sporting arena include open parking, parking facility size, and inadequate lighting (Clarke 2002).

Mischief had different spatial patterns for game and comparison days. This crime type is typically referring to property damage such as vandalism. Crime patterns showed that for home games and comparison away games there were increases in the Downtown area, particularly the northwestern portion close to English Bay. Examining hockey fan behaviour during home games may prove to be an interesting sociological study, similar to the football hooliganism studies in the United Kingdom (Dunning et al. 2014), where stadiums act as crime attractors or generators (Brantingham and Brantingham 1981). We also considered residential burglary, commercial burglary, and theft‐of‐vehicle crime types for GWR models. Twitter activity showed a poor influence for these crime types; however, neither of the other explanatory variables were significant enough to generate a good model. As such, it is important to note the social media data may not be instructive for all crime types in all contexts.

Despite these interesting results, our study is not without limitations. The first is related to the availability of only six crime types from the Vancouver Open Data Catalogue. The information freely available from the Vancouver Police Department does not reflect the total number of incidents, specifically violent crime types. Future research should analyze other crime types, such as assaults, violence against the person, robberies, or pickpocketing that have been shown to be related with sport events in the literature, but not precisely with hockey games (Kurland 2014; Marie 2016; Montolio and Planells 2016). The second limitation is the ever‐present modifiable areal unit problem (Openshaw 1984). Future work should consider different scales for this study area and emphasize the differences, if any, in the explanatory variables’ statistical significance. Also, another issue mentioned above is the potential bias and issues with limited access for Twitter data, which could be overcome by using an additional social media dataset.

Overall, our analyses presented spatial regression models for spatial crime analysis using common explanatory variables such as residential population, parks, public roads, liquor stores, and others. The dynamic variable included in the models was Twitter data, included first as a geolocated messages dataset and second as crime‐tweets subset. This study emphasizes the influence of all these variables on crime models, specifically for hockey games’ temporal frame and their comparison days in Vancouver. Having now tested the implications of crime‐tweets in these global and local regression models, it is our intent to develop our analysis in multiple ways, including cascading latent Dirichlet allocation (LDA) (Blei et al. 2003) for topic modelling and identifying types of violent topics in the tweets, and then applying sentiment analysis and term frequency analysis. Also, a spatiotemporal LDA is being considered, which would define more detailed connections between violent tweets and crime locations, if any. This study has illustrated that Twitter data, supplemented by additional contextual information, can be useful in shedding light on the geography of crime and may prove helpful in predicting future crime occurrences.
